# How Does a 12-Week Physical Exercise Program Affect the Motor Proficiency and Cognitive Abilities of Overweight and Normal-Weight Preschool Children?

**DOI:** 10.3390/children11040479

**Published:** 2024-04-17

**Authors:** Borko Katanic, Aleksandra Aleksic Veljkovic, Radivoje Radakovic, Nenad Stojiljkovic, Mihai Olanescu, Miruna Peris, Adrian Suciu, Danut Popa

**Affiliations:** 1 Faculty of Sport and Physical Education, University of Nis, 18000 Nis, Serbia; borkok@ucg.ac.me (B.K.); aleksandra.aleksic.veljkovic@fsfv.ni.ac.rs (A.A.V.); nenad@fsfv.ni.ac.rs (N.S.); 2 Institute for Information Technologies, University of Kragujevac, Jovana Cvijića bb, 34000 Kragujevac, Serbia; radivoje.radakovic@pfu.kg.ac.rs; 3 Faculty of Pedagogy, University of Kragujevac, Trg Svetog Save 36, 31000 Uzice, Serbia; 4 Faculty of Automotive, Mechatronics and Mechanical Engineering, Technical University of Cluj-Napoca, 400641 Cluj-Napoca, Romania; adrian.suciu@mdm.utcluj.ro (A.S.); ilie.popa@mdm.utcluj.ro (D.P.); 5 Faculty Industrial Engineering, Robotics and Production Management, Technical University of Cluj-Napoca, 400641 Cluj-Napoca, Romania; miruna.peris@muri.utcluj.ro

**Keywords:** preschoolers, obesity, motor abilities, cognitive functions, BMI, TGMD-2, BOT-2

## Abstract

The objective of this research was to examine a 12-week exercise program and its impact on the motor proficiency and cognitive abilities of preschool children with overweight and normal weight. The study involved a total of 71 participants who were preschool children enrolled in a longitudinal study. Body mass index (BMI) was determined by measuring body height and weight, and the nutritional status of the children was assessed using the World Health Organization’s (WHO) criteria. Motor proficiency encompasses both motor abilities and motor skills, and the assessment of motor abilities was conducted using subtests from the Bruininks–Oseretsky Test of Motor Proficiency (BOT-2). These subtests measured fine motor integration, manual dexterity, balance, and bilateral coordination. The evaluation of motor skills involved the utilization of the Test of Gross Motor Development (TGMD-2), which examines both locomotor skills and manipulative skills. Cognitive abilities were assessed using the School Maturity Test (TZS). All participants, regardless of weight status, took part in a 12-week physical exercise program. According to the World Health Organization’s criteria, 52 children (73.2%) were categorized as having a normal weight, while 19 children (26.8%) were classified as overweight. These findings indicate that every fourth child in the study was overweight. Using a statistical analysis called SPANOVA (2 × 2, group × time), differences were observed in three out of eleven variables. Specifically, there were significant differences in two motor skill variables: manipulative skills (*p* = 0.006) and total movement skills (*p* = 0.014). Additionally, there was a significant difference in one cognitive ability variable: visual memory (*p* = 0.010). No significant differences were found in the remaining variables. The findings of this study aimed to contribute to the understanding of the potential benefits of regular exercise on motor and cognitive development in preschool children, specifically examining the differences between overweight and normal-weight children. By investigating these effects, the study could provide valuable insights for educators, parents, and health professionals involved in promoting the overall well-being of preschool-aged children. Regular physical exercise has been found to have positive effects on motor and cognitive abilities in both overweight and normal-weight preschool children.

## 1. Introduction

The influence of modern lifestyle has led to children spending increasingly more time sitting while playing video games or surfing the internet and having less time for physical activities. According to Colley et al. [1], children today spend up to half of their waking hours in a seated position. A lack of physical activity (hypokinesia), combined with a sedentary lifestyle and malnutrition, contributes to childhood obesity [2,3]. This condition is linked to various health complications, including type 2 diabetes, asthma, hypertension, early atherosclerosis, and psychosocial challenges [4].

Childhood obesity has become increasingly prevalent, emerging as a significant epidemiological problem and a major challenge to public health [5]. Recent studies involving large samples of children have demonstrated a notable trend of increased rates of overweight and obesity over the past two decades [6,7,8,9,10]. 

The World Health Organization (WHO) points out that the prevalence of overweight and obesity in the world has dramatically risen in recent decades, highlighting that in 1975, the prevalence of obesity among children was only 4%, whereas according to recent data, it has reached as high as 18%. Additionally, it is estimated that today, the number of obese children and adolescents between the ages of 5 and 19 has surpassed 340 million [11]. Research by Spiotta and Luma [12] confirms previous information on the prevalence of obesity, highlighting that every third child is overweight. These recent trends in childhood obesity are indeed alarming, prompting active involvement from the World Health Organization (WHO) in addressing this issue. In response, WHO has adopted a strategy called the Global Strategy on Diet, Physical Activity, and Health [13].

A particular challenge lies in the high body weight and excess weight among preschool children, as this period lays the foundation for the development and prediction of obesity in later years (6–19 years) [12]. The body mass index (BMI) in children during the preschool period is linked to markers of cardiovascular disease risk [13] and has been recognized as a contributing element to increased BMI during childhood and adolescence, leading to a higher risk of health problems [12,13]. High body weight and excess weight among preschool children have become a growing global concern [14,15]. This is particularly worrisome as early childhood is well known for its crucial role in predicting obesity in adolescence [16,17]. BMI in preschool children is linked to cardiovascular disease risk markers [18] and serves as an indicator for higher BMI during childhood and later adolescence, posing a risk factor for health issues [16,19]. Therefore, it is crucial to recognize and address the issue of obesity in a timely manner to ensure healthier childhoods and futures for all generations. This requires integrating physical activities into children’s daily lives, as well as promoting healthy eating habits and limiting screen time, in line with the WHO strategy [13]. 

At the preschool age, the proper motor and cognitive functioning of children is very important, so there are increasingly more studies addressing this area [20,21,22]. Motor proficiency includes both motor abilities and motor skills. Motor abilities represent the latent abilities of a person involved in performing motor activities [23], while motor skills refer to the manner of performing locomotor and manipulative motor tasks [24]. On the other hand, cognitive abilities are understood as mental actions or processes of acquiring knowledge and understanding through thought, experience, and senses [25].

Researchers have identified a negative impact of high body mass percentage and obesity on motor skills [26,27]. Recent studies have shown a negative correlation between excessive BMI and basic motor abilities [28,29]. It is believed that children with excess weight develop motor skills at a slower pace in comparison to their normal-weight peers [30]. A key concern is that obese children frequently abstain from physical activities, which can exacerbate the deterioration of their motor skills and contribute to further weight gain [26].

Some authors have highlighted a negative connection between obesity and cognitive functions [31], and there is evidence of a potential link between overweight and poor cognitive function [32]. Recent studies have demonstrated a connection between excessive weight and intelligence or the general impairment of cognitive abilities, as well as associations with various cognitive domains [32,33,34].

Some research on preschool-aged children indicates that the physical exercise program influences motor abilities, primarily coordination and speed–strength abilities [35,36]. Meanwhile, some authors also point out the positive effects of physical education intervention on certain cognitive abilities of children [37]. Although to our knowledge there are no studies examining the impact of physical exercise intervention on obese preschool-aged children, studies of this type at a slightly older age in children and adolescents should still be considered. It should be noted that exercise programs can lead to significant improvements in body composition [38], as well as psychological and motor qualities in children and adolescents [39]. On the other hand, there have been extensive studies exploring the motor and cognitive domains and differences between normally nourished and obese children [34,40], but there is a lack of longitudinal research examining whether specific exercise programs have differential effects on the motor and cognitive status of normally nourished and obese children. In this regard, the aim of this study was to examine how a 12-week exercise program impacts the motor proficiency and cognitive abilities of preschoolers with overweight and normal weight. Based on this aim, the following hypothesis was formulated: there is a significant difference in the effects of physical exercise programs on motor and cognitive status between normally nourished and overweight preschool children. This research can contribute to a deeper understanding of the impact of physical exercise programs on key components of motor development, such as motor and cognitive abilities, in children of that age group. Additionally, its significance lies in being one of the rare studies examining intervention programs for obese preschool children, which can provide significant findings.

## 2. Materials and Methods

### 2.1. Participants

A longitudinal study was conducted with a sample of 71 children, aged 5 and 6, who were selected randomly from the preschool institution in Požarevac (Serbia). The children were divided according to BMI into two groups: normal weight (n = 52, age 6.09 ± 0.39 years) and overweight (n = 19, age 6.30 ± 0.36 years). For each participant, their age in years and months (decimal values) was calculated. In order to participate in the study, the children had to fulfill specific requirements: they had to be in good health, free from any illnesses or disorders, of both genders, between the ages of five and six years, and not engaged in any sports activities outside the scope of the preschool curriculum.

All parents of the participants are informed about the aim of the research and have provided written consent, confirming that their children can participate in this study. The research was conducted in accordance with the principles of the Helsinki Declaration and approved by the Ethics Committee of the Faculty of Sport and Physical Education in Niš (Ref. No. 04-1186/2).

### 2.2. Anthropometric Characteristics

Anthropometric characteristics were assessed using standardized anthropometric instruments following established international protocols [41]. BMI, as a measure of nutritional status, was calculated using the standard formula: BMI = BM (kg)/BH (m)^2^, where BM represents body mass and BH represents body height. BMI serves as a reliable indicator of weight status in children, as it shows a strong correlation with body fat content [42].

Based on their BMI status, the children were classified into two groups: Healthy (normal) weight and overweight, as presented in Table 1. Children are classified according to WHO cutoff points, which are determined according to gender and age, divided into 3-month intervals [43]. So, for each child, their BMI is measured according to their exact chronological age and gender, and based on that, the group to which they belong is determined.

### 2.3. Motor Abilities (BOT-2) 

The assessment of motor abilities was conducted using specific subtests from the Bruininks–Oseretsky Test (BOT-2). The BOT-2 test consists of eight subtests that assess motor abilities such as fine motor precision, fine motor integration, manual dexterity, bilateral coordination, balance, running speed and agility, upper-limb coordination, and strength. This test is a widely recognized standardized tool for evaluating the motor ability levels of children [44]. Previous research in this field has demonstrated the validity of the BOT-2 test in a sample of children aged 4 to 21 years [45].

For the purpose of this study, four specific subtests from the BOT-2 test battery were utilized. Through 29 motor tasks, the following motor abilities were examined: fine motor integration (consisting of 8 motor tasks), manual dexterity (5 tasks), balance (9 tasks), and bilateral coordination (7 tasks). The last two subtests, balance and bilateral coordination, contributed to the assessment of overall body coordination, providing additional insights into the examinee’s coordination ability.

### 2.4. Motor Skills (TGMD-2) 

In order to assess basic motor skills, the Test of Gross Motor Development-2 (TGMD-2) was utilized. This test employs an observational technique to evaluate children’s motor skills. The TGMD-2 has been validated in a sample of children aged from 3 to 12 years, thus encompassing the preschool age range [46]. It consists of 12 motor tasks divided into two subtests: locomotor and manipulative movement skills. Locomotor skills are assessed based on six motor tasks such as running, jumping, and so on, while manipulative skills involve six object control tasks such as throwing a ball, catching, kicking with the foot, etc. Locomotor and manipulative skills collectively contribute to a total gross motor score. This total score represents the overall level of motor skills in children.

### 2.5. Cognitive Abilities (TZS)

Children’s cognitive abilities were assessed using the School Maturity Test (ser. Test Zrelosti za školu—TZS). This test battery was designed to allow psychologists to examine children’s cognitive functioning before they start school. Specifically, through five subtests, the SMT covers cognitive abilities such as memory, attention, visual–motor coordination, planning ability, perceptual organization, and others [47]. In a study [48] that examined the metric characteristics of the SMT on children aged five to seven years, the test showed high reliability and validity. It is also worth noting that the SMT demonstrated a strong correlation with the TIP-1 tests and Raven’s Coloured Progressive Matrices [48].

In this particular study, three subtests were used. Each test assesses several cognitive abilities. The Visual Memory Test evaluates children’s memory and attention through 15 cognitive tasks. Using the Stacking Cubes Test (15 tasks), psychologists assess the visual–motor coordination, planning ability, and perceptual organization of the participants. Meanwhile, the Codes Test (8 tasks) evaluates cognitive capacities related to learning from experience, concentration, and visual–motor coordination.

The obtained results from all three tests were then converted using standardized SMT tables with cutoff values. The authors of this test provide an SMT table that is standardized according to the participants’ gender and their decimal age in 3-month increments. Upon the completion of conversion, the standardized final values underwent further analysis in this study.

### 2.6. Variables

The total score for each subtest was recorded and subsequently converted based on the standardized tables provided by the authors of the BOT-2 [49], TGMD-2 [46], and TZS tests [47]. As a result, the standardized data were entered for subsequent analysis, resulting in six variables related to motor abilities, three variables related to motor skills, and four variables associated with cognitive abilities.

### 2.7. Experimental Program

The program was developed according to the guidelines provided by the National Association for Sport and Physical Education (NASPE; USA), which recommends a combination of aerobic and strength exercises for children in this age group [50]. The Physical Education Intervention (PEI) consisted of aerobic and strength exercises and was implemented three times a week for a duration of 30 min over a period of 12 weeks. Each session comprised three components: a 5 min warm-up phase, a 20 min main phase, and a 5 min cool-down phase.

During the main phase, typically, 8 to 10 exercise complexes were included, focusing on bodyweight exercises adapted for children. The cool-down phase lasted approximately 5 min and involved stretching exercises. The PEI sessions were conducted by trained Physical Education (PE) teachers.

### 2.8. Procedure

All the testing in this longitudinal study, both initial and final, was conducted at the main kindergarten facility in Požarevac. Motor proficiency tests were carried out in the kindergarten’s hall. The tests were conducted simultaneously (at 11 a.m.) to exclude daily variations in measurements. The room temperature during testing ranged from 22 °C to 26 °C. Motor proficiency was assessed by two researchers (authors B.K. and A.A.V.), holding doctoral degrees in physical education and sports, and experienced in conducting and evaluating motor tasks. Children were assessed together, with any discrepancies resolved by consensus. It took approximately 30 min per participant to assess motor tasks. The initial testing was conducted in November 2021, and the final testing took place in January 2022, i.e., after 12 weeks of intervention.

Cognitive ability tests were conducted on the premises of the preschool institution. Measures were taken to ensure that participants entered the psychologist’s office one by one, affording each child better attention while solving tasks and preventing other children from observing the solution of cognitive tasks. Cognitive tests were administered by two psychologists holding doctoral and master’s degrees in psychological sciences. Both psychologists have extensive experience in testing children. They conducted cognitive tests and interpreted the results, in accordance with Article 10 of the Regulations on the use of psychological measurement instruments [51].

### 2.9. Statistics

Basic central and distribution parameters were calculated for all data obtained through testing, including the mean and standard deviation. To evaluate the effects of the experimental program, a mixed-design ANOVA 2 × 2 model (SPANOVA group × time) was employed. This statistical analysis allowed for the examination of both between-group (group factor) and within-group (time factor) differences. A significance level of *p* < 0.05 was adopted for the study. All data analysis procedures were performed using IBM SPSS Statistics 26 software, which is used in social sciences (SPSS v26.0, SPSS Inc., Chicago, IL, USA). 

## 3. Results

Table 2 displays the distribution of the sample according to gender and BMI, revealing that there are 52 (73.2%) children classified as normally nourished and 19 (26.8%) children classified as obese. A similar proportion of normally nourished and obese children is observed for both girls and boys. This indicates that approximately one in every four participants is obese.

Table 3 presents the results of all motor and cognitive tests at the initial and final measurements for both groups of participants. Using mixed ANOVA (SPANOVA, group × time), significant differences were found in three out of the thirteen variables. Specifically, significant differences were observed in two motor skill variables: manipulative skills (*p* = 0.006) and total movement skills (*p* = 0.014), as well as in one cognitive ability variable: visual memory (*p* = 0.010). No significant differences were found in the remaining variables.

## 4. Discussion

The aim of the present study was to explore how a twelve-week exercise intervention impacts the motor proficiency and cognitive abilities of preschool children with overweight and normal weight. Descriptive statistics revealed that there are 52 (73.2%) children classified as normally nourished and 19 (26.8%) children classified as obese, and ANOVA results showed significant differences between these groups in two motor skill variables: manipulative skills (*p* = 0.006) and total movement skills (*p* = 0.014), as well as in one cognitive ability variable: visual memory (*p* = 0.010). No significant differences were found in the remaining variables. The values of η^2^ did not reach Cohen’s [52] limit of practical significance (η^2^ = 0.14); this means that a strong relationship between the given variables was not achieved.

We have hypothesized that there is a significant difference in the effects of physical exercise programs on motor and cognitive status between normal-weight and overweight preschool children, but the program did not have statistically significant differences in effects between groups on fine motor integration, manual dexterity, and body coordination. The results of descriptive statistics showed that obese children had lower results on the final measurement compared to the initial. Another variable in which the results after 12 weeks of practice in the overweight group were lower is visual memory. It is certain that further research is needed on a larger sample of respondents in order to establish the reasons for such results. Although there was no difference in effects between the groups, it is evident that in other variables based on descriptive statistics, there was progress in both groups and that the program was effective. Increasing the duration of the experimental treatment in future research could be a valid approach to exploration. A longer period of time might provide a prospect for participants to fully engage with the intervention, leading to greater effects on the primary outcome or other variables of interest. However, it is essential to consider potential factors such as participant compliance, feasibility, and potential dropout rates over an extended period. Although, it should be noted that in a large number of studies, when cognitive abilities were observed through a single variable representing the overall factor of cognitive abilities, positive effects were achieved in the majority of studies [53,54,55,56]. Contrary to this, there were studies in which physical exercise interventions did not yield significant effects on cognitive abilities [57,58]. A review study by Zeng et al. [59] also indicates the need for more research, as well as research on a larger sample of preschool children. The aim of the research was to consolidate studies on the effects of physical activity on motor skills and cognitive development in healthy preschool children, as well as to provide a synthesis of current evidence regarding their association and effects. Fifteen studies were included in the analysis, concluding that increased physical activity has significant beneficial effects in 80% of studies evaluating motor skills and cognitive development. Importantly, there are no studies where the increased duration or frequency of physical activity had significant harmful effects on motor skills and cognitive development in young children.

Venetsanou and Kambas [60] emphasized that children with lower scores on motor proficiency should be provided with plenty of opportunities to access developmentally appropriate movement interventions that could help them overcome or minimize their motor development delays by targeting specific areas of improvement. The authors recommended that programs should be designed to meet the individual requirements of each child and provide them with appropriate challenges and support, so we tried to fulfill those criteria in our study. The collected data indicate that the implementation of intervention programs can have a positive impact on improving motor competence [61] as well as psychomotor profiles [62] in preschoolers. Battaglia et al. [63] reported that the physical education program that they conducted showed effectiveness in improving motor and pre-literacy skills in preschool children regardless of their age and gender. They also observed that weight status did not influence these skills, suggesting that the program can be equally applied to children with different weight categories. Therefore, taking into account our results, the conclusion is that the program can be successful in both cases in enhancing motor and cognitive development in preschool children and achieving positive outcomes.

Roberts et al. [64] noticed conflicting findings in previous studies when it comes to the relationship between motor skills and weight status in preschool-aged children and this is because it is complex and may be influenced by various factors, but we did not find a single study that aimed to investigate the impact of the program on overweight and normal-weight children. It is possible that individual differences, sample characteristics, measurement methods, or other contextual factors contribute to these discrepancies [64]. Their study results indicated a connection between obesity and gross motor skills, where preschoolers with poorer gross motor skills struggle with tasks related to locomotion and body stabilization. So, we tried to include all necessary variables that can be important for the proper motor development of children involved in our study. In accordance with earlier studies, 12 weeks of intervention has a positive effect on the transformation of motor and cognitive abilities [65], and those authors stated that physical education teachers and other educators working with children should engage them, with a particular emphasis on the type and frequency of physical activities that would enable children to develop motor skills and, thus, enhance the motor development of preschoolers.

The biggest limitation of our study is the small sample, so the children were divided into only two categories. Earlier studies showed children who were classified as overweight jumped further and hopped longer than children in the obese category [64], but in ours, there was no possibility for such comparisons, or for the examination of differences between the sexes that were studied in the majority of earlier studies [60,64]. Another limitation concerns the fact that BMI is an indicator of weight status, but not an indicator of body composition. For future research, it would be advisable to include, in addition to anthropometric parameters, an analysis of body composition that will provide additional information about the distribution of fat and muscle mass in preschool children. This will provide more precise information about the percentage of body fat in each child. Based on reference values for body fat according to children’s age, more accurate information about the child’s weight status will be obtained. Consequently, the groups will be more precisely categorized accordingly.

Despite the mentioned limitations, this study is highly significant as it is one of the few to compare the effects of a 12-week training program on motor and cognitive abilities between obese and normally nourished preschool children. This is especially true considering the scarcity of studies addressing the treatment of obese preschoolers, and moreover, it is very rare for a study in this age group to encompass both the motor and cognitive domains of children. Therefore, this study can be highly valuable and provide guidance that will assist researchers in further investigations.

## 5. Conclusions

The aim of this research was to examine how a twelve-week exercise program affects the motor and cognitive abilities of preschoolers with overweight and normal weight. Based on the results, significant differences were observed in two motor skill variables: manipulative skills and overall motor skills, as well as in one cognitive ability variable: visual memory. However, there were no differences among groups in the effects of intervention in other motor and cognitive variables.

The findings of this study aimed to contribute to the understanding of the potential benefits of regular exercise on motor and cognitive development in preschool children, by specifically examining the differences between overweight and normal-weight children. By investigating these effects, the study could provide valuable insights for educators, parents, and health professionals involved in promoting the overall well-being of preschool-aged children. Regular physical exercise has been found to have positive effects on motor and cognitive abilities in both overweight and normal-weight preschool children. Therefore, these findings indicate the need for interventions in early childhood physical exercise as a key factor in promoting health and development in preschool children. Additionally, these findings can serve as a basis for the development of public policies aimed at enhancing physical activity among preschool children.

## Figures and Tables

**Table 1:** Descriptive statistics (Mean ± SD).

	Healthy (n = 52)	Overweight (n = 19)
Age	6.09 ± 0.39	6.30 ± 0.36
Body weight (kg)	21.39 ± 2.44	27.78 ± 5.64
Body height (m)	1.18 ± 0.05	1.21 ± 0.07
BMI (kg/m^2^)	15.28 ± 1.07	18.83 ± 2.56

Note: mean ± standard deviation.

**Table 2:** Distribution of the sample according to gender and BMI.

	Boys (33) (46.5%)	Girls (38) (53.5%)	Total (71) (100%)
Healthy weight	24 (72.7%)	28 (73.7%)	52 (73.2%)
Overweight	9 (27.3%)	10 (26.3%)	19 (26.8%)

**Table 3:** Motor and Cognitive Test Results at Baseline and After Intervention.

Variables	Healthy Weight		Overweight		*p*	ηp^2^
	Initial	Final	Initial	Final		
Fine motor integration	13.42 ± 4.09	13.44 ± 2.88	13.79 ± 4.42	12.95 ± 3.98	0.423	0.009
Manual dexterity	12.13 ± 4.17	15.29 ± 5.52	11.58 ± 4.60	12.58 ± 5.57	0.153	0.029
Fine motor skills	25.56 ± 6.35	28.73 ± 6.98	25.37 ± 7.53	25.53 ± 8.47	0.079	0.044
Bilateral coordination	14.44 ± 2.93	17.65 ± 2.86	15.05 ± 2.55	17.21 ± 2.86	0.155	0.029
Balance	14.02 ± 4.41	15.69 ± 4.76	12.42 ± 4.59	14.53 ± 5.08	0.681	0.002
Body coordination	28.46 ± 6.19	33.35 ± 5.85	27.47 ± 6.16	31.74 ± 7.50	0.639	0.003
Locomotor skills	6.88 ± 1.61	7.77 ± 1.86	6.21 ± 1.58	7.58 ± 1.57	0.240	0.020
Manipulative skills	7.65 ± 1.74	7.92 ± 2.01	6.42 ± 1.12	8.16 ± 2.06	0.006 *	0.106
Total movement skills	14.54 ± 3.02	15.69 ± 3.63	12.63 ± 2.24	15.74 ± 3.35	0.014 *	0.085
Visual memory	3.10 ± 0.57	3.14 ± 0.49	3.42 ± 0.60	3.00 ± 0.00	0.010 *	0.093
Stacking cubes	4.23 ± 0.85	4.29 ± 0.75	4.05 ± 0.97	4.05 ± 0.78	0.803	0.001
Code	3.31 ± 0.81	3.79 ± 0.80	3.00 ± 1.00	3.74 ± 0.56	0.278	0.017
Total cognitive abilities	3.55 ± 0.57	3.74 ± 0.54	3.49 ± 0.57	3.60 ± 0.39	0.490	0.007

Note: mean ± standard deviation; p-coefficient of the significance of the differences; * at the *p* < 0.05 level; ηp^2^-partial Eta squared.

## Data Availability

Data will be provided to all interested parties upon reasonable request. The data are not publicly available due to privacy considerations.

## References

[1] Colley R.C., Garriguet D., Adamo K.B., Carson V., Janssen I., Timmons B.W., Tremblay M.S. (2013). Physical activity and sedentary behavior during the early years in Canada: A cross sectional study. Int. J. Behav. Nutr. Phys. Act..

[2] Planinsec J., Matejek C. (2004). Differences in physical activity between non-overweight, overweight and obese children. Coll. Antropol..

[3] Mendonça C.P., Anjos L.A. (2004). Dietary and physical activity factors as determinants of the increase in overweight/obesity in Brazil. Cad. Saude Publica.

[4] Dikanović V., Vignjević Z. (2009). Gojaznost dece uzrasta 7 do 15 godina i rizik za pojavu dijabetes mellitusa tipa 2 (Obesity in children aged 7 to 15 years and the risk of type 2 diabetes mellitus). U Živić. S. (Ur.). 42. Pedijatrijski Dani Srbije Sa Međunarodnim Učešćem.

[5] Kumar S., Kaufman T. (2018). Childhood obesity. Panminerva Med..

[6] Wang Y., Lobstein T.I.M. (2006). Worldwide trends in childhood overweight and obesity. Pediatr. Obes..

[7] Ogden C.L., Carroll M.D., Lawman H.G., Fryar C.D., Kruszon-Moran D., Kit B.K., Flegal K.M. (2016). Trends I obesity prevalence among children and adolescents in the United States, 1988–1994 through 2013–2014. JAMA.

[8] Abarca-Gómez L., Abdeen Z.A., Hamid Z.A., Abu-Rmeileh N.M., Acosta-Cazares B., Acuin C., Agyemang C., Adams R.J., Aekplakorn W., Afsana K. (2017). Worldwide trends in body-mass index. Underweight, overweight, and obesity from 1975 to 2016: A pooled analysis of 2416 population-based measurement studies in 128· 9 million children, adolescents and adults. Lancet.

[9] Skinner A.C., Ravanbakht S.N., Skelton J.A., Perrin E.M., Armstrong S.C. (2018). Prevalence of obesity and severe obesity in US children, 1999–2016. Pediatrics.

[10] Boddy L.M., NCD Risk Factor Collaboration (2020). Height and body-mass index trajectories of school-aged children and adolescents from 1985 to 2019 in 200 countries: Pooled analysis of 2.182 population-based studies with 65 million participants. Lancet.

[11] World Health Organization Report of the Commission on Ending Childhood Obesity. https://www.who.int/publications/i/item/9789241510066.

[12] Spiotta R.T., Luma G.B. (2008). Evaluating obesity and cardiovascular risk factors in children and adolescents. Am. Fam. Physician.

[13] World Health Organization Obesity and Overweight. https://www.who.int/news-room/fact-sheets/detail/obesity-and-overweight.

[14] de Onis M., Blössner M., Borghi E. (2010). Global prevalence and trends of overweight and obesity among preschool children. Am. J. Clin. Nutr..

[15] Starc G., Popović S., Đordić V., Ostojić S., Musić Milanović S., Kujundžić E., Spiroski I., Đurić S., Mašanović B., Sember V. (2019). Differences in body height between the contemporary Western Balkan children and the WHO growth references core sample. Anthropol. Noteb..

[16] Reilly J.J., Kelly J. (2011). Long-term impact of overweight and obesity in childhood and adolescence on morbidity and premature mortality in adulthood: Systematic review. Int. J. Obes..

[17] Lundeen E.A., Norris S.A., Adair L.S., Richter L.M., Stein A.D. (2016). Sex differences in obesity incidence: 20-year prospective cohort in South Africa. Pediatr. Obes..

[18] Messiah S.E., Arheart K.L., Natale R.A., Hlaing W.M., Lipshultz S.E., Miller T.L. (2012). BMI, waist circumference, and selected cardiovascular disease risk factors among preschool-age children. Obesity.

[19] Ekelund U., Luan J., Sherar L.B., Esliger D.W., Griew P., Cooper A. (2012). International Children’s Accelerometry Database C. Moderate to vigorous physical activity and sedentary time and cardiometabolic risk factors in children and adolescents. JAMA.

[20] Hernandez A.M., Caçola P. (2015). Motor proficiency predicts cognitive ability in four-year-olds. Eur. Early Childhood Educ. Res. J..

[21] Van der Fels I.M., Te Wierike S.C., Hartman E., Elferink-Gemser M.T., Smith J., Visscher C. (2015). The relationship between motor skills and cognitive skills in 4–16 year old typically developing children: A systematic review. J. Sci. Med. Sport.

[22] Banjevic B., Aleksic D., Aleksic Veljkovic A., Katanic B., Masanovic B. (2022). Differences between Healthy-Weight and Overweight Serbian Preschool Children in Motor and Cognitive Abilities. Int. J. Environ. Res. Public Health.

[23] Malacko J., Popović D. (2001). Metodologija Kineziološko Antropoloških Istraživanja, III Izdanje (Methodology of Kinesiological Anthropological Research).

[24] Magill R.A., Anderson D. (2010). Motor Learning and Control.

[25] Davis E.E., Pitchford N.J., Limback E. (2011). The interrelation between cognitive and motor development in typically developing children aged 4–11 years is underpinned by visual processing and fine manual control. Br. J. Psychol..

[26] D’Hondt E., Deforche B., De Bourdeaudhuij I., Lenoir M. (2009). Relationship between motor skill and body mass index in 5- to 10-year-old children. Adapt. Phys. Activ. Q..

[27] Gentier I., D′Hondt E., Shultz S., Deforche B., Augustijn M., Hoorne S., Verlaecke K., De Bourdeaudhuij I., Lenoir M. (2013). Fine and gross motor skills differ between healthy-weight and obese children. Res. Dev. Disabil..

[28] D′Hondt E., Deforche B., Gentier I., Verstuyf J., Vaeyens R., De Bourdeaudhuij I., Philippaerts R., Lenoir M. (2014). A longitudinal study of gross motor coordination and weight status in children. Obesity.

[29] Barnett L.M., Lai S.K., Veldman S.L., Hardy L.L., Cliff D.P., Morgan P.J., Zask A., Lubans D., Shultz S., Ridgers N. (2016). Correlates of gross motor competence in children and adolescents: A systematic review and meta-analysis. Sports Med..

[30] Nervik D., Martin K., Rundquist P., Cleland J. (2011). The relationship between body mass index and gross motor development in children aged 3 to 5 years. Pediatr. Phys. Ther..

[31] Dye L., Boyle N.B., Champ C., Lawton C. (2017). The relationship between obesity and cognitive health and decline. Proc. Nutr. Soc..

[32] Guardabassi V., Tomasetto C. (2020). Weight status or weight stigma? Obesity stereotypes—Not excess weight—Reduce working memory in school-aged children. J. Exp. Child Psychol..

[33] Prickett C., Brennan L., Stolwyk R. (2015). Examining the relationship between obesity and cognitive function: A systematic literature review. Obes. Res. Clin..

[34] de Waal E., Pienaar A.E. (2021). Influences of persistent overweight on perceptual-motor proficiency of primary school children: The North-West CHILD longitudinal study: Persistent overweight and perceptual-motor proficiency in children. BMC Pediatr..

[35] Krneta Ž., Casals C., Bala G., Madić D., Pavlović S., Drid P. (2015). Can kinesiological activities change “Pure” motor development in preschool children during one school year?. Coll. Antropol..

[36] Birnbaum J., Geyer C., Kirchberg F., Manios Y., Koletzko B., ToyBox-study Group (2017). Effects of a kindergarten-based, family-involved intervention on motor performance ability in 3-to 6-year-old children: The ToyBox-study. J. Sports Sci..

[37] Fisher A., Boyle J.M., Paton J.Y., Tomporowski P., Watson C., McColl J.H., Reilly J.J. (2011). Effects of a physical education intervention on cognitive function in young children: Randomized controlled pilot study. BMC Pediatr..

[38] Fanelli E., Abate Daga F., Pappaccogli M., Eula E., Astarita A., Mingrone G., Veglio F. (2022). A structured physical activity program in an adolescent population with overweight or obesity: A prospective interventional study. Appl. Physiol. Nutr. Metab..

[39] Schranz N., Tomkinson G., Parletta N., Petkov J., Olds T. (2014). Can resistance training change the strength, body composition and self-concept of overweight and obese adolescent males? A randomised controlled trial. Br. J. Sports Med..

[40] Li Y., Dai Q., Jackson J.C., Zhang J. (2008). Overweight is associated with decreased cognitive functioning among school-age children and adolescents. Obesity.

[41] Eston R., Reilly T. (2013). Kinanthropometry and Exercise Physiology Laboratory Manual: Tests, Procedures and Data: Volume Two: Physiology.

[42] Wilmore J.H., Costill D.L., Kenney W.L., Wilmore J., Costill D.L. (2008). Body composition in sport. Physiology of Sport and Exercise.

[43] Onis M.D., Onyango A.W., Borghi E., Siyam A., Nishida C., Siekmann J. (2007). Development of a WHO growth reference for school-aged children and adolescents. Bull. World Health Organ..

[44] Deitz J.C., Kartin D., Kopp K. (2007). Review of the Bruininks-Oseretsky Test of Motor Proficiency, Second Edition (BOT-2). Phys. Occup. Ther. Pediatr..

[45] Abbas J., Tedla J.S., Krishnan S.K. (2011). Normative Data for Bruininks-Oseretsky Test of Motor Proficiency (BOTMP) in Children of 9½–14½ years: A Cross-Sectional Study. Crit. Rev. Phys. Rehabil. Med..

[46] Ulrich R.S. (2000). Evidence based environmental design for improving medical outcomes. Proceedings of the Healing by Design: Building for Health Care in the 21st Century Conference.

[47] Novović Z., Biro M., Baucal A., Tovilović S. (2007). Test Zrelosti za Školu (Maturity Test for School).

[48] Novović Z., Tovilović S., Jovanović V., Biro M. (2009). Validacija testa zrelosti za školu (Validation of maturity test for school). Primenj. Psihol..

[49] Bruininks R.H., Bruininks B.D. (2005). BOT2: Bruininks-Oseretsky Test of Motor Proficiency: Manual.

[50] US Department of Health and Human Services (USDHHS) (2008). Physical Activity Guidelines for Americans.

[51] Društvo psihologa Srbije Pravilnik o Standardima i Procedurama Izrade i Upotrebe Psiholoških Mernih Instrumenata (Rulebook on Standards and Procedures for the Creation and Use of Psychological Measuring Instruments). https://www.dps.org.rs/2012/794/?_rstr_nocache=rstr166661e5acc6733d.

[52] Cohen J. (1988). Statistical Power Analysis for the Behavioral Sciences.

[53] Badiei M., Sulaiman T. (2014). The Difference between Montessori Curriculum and Malaysia National Preschool Curriculum on Developmental Skills of Preschool Children in Kuala Lumpur. J. Educ. Soc. Behav. Sci..

[54] Morse M.N. (2017). Physical Activity in the Preschool Classroom: An Approach to Enhance Executive Functioning through the Move for Thought Prek-k Program. Master′s Thesis.

[55] Xiong S., Li X., Tao K. (2017). Effects of Structured Physical Activity Program on Chinese Young Children’s Executive Functions and Perceived Physical Competence in a Day Care Center. Biomed. Res. Int..

[56] Xiong S., Zhang P., Gao Z. (2019). Effects of Exergaming on Preschoolers’ Executive Functions and Perceived Competence: A Pilot Randomized Trial. J. Clin. Med..

[57] Wen X., Zhang Y., Gao Z., Zhao W., Jie J., Bao L. (2018). Effect of Mini-Trampoline Physical Activity on Executive Functions in Preschool Children. Biomed. Res. Int..

[58] Jaksic D., Mandic S., Maksimovic N., Milosevic Z., Roklicer R., Vukovic J., Drid P. (2020). Effects of a Nine-Month Physical Activity Intervention on Morphological Characteristics and Motor and Cognitive Skills of Preschool Children. Int. J. Environ. Res. Public Health.

[59] Zeng N., Ayyub M., Sun H., Wen X., Xiang P., Gao Z. (2017). Effects of Physical Activity on Motor Skills and Cognitive Development in Early Childhood: A Systematic Review. Biomed. Res. Int..

[60] Venetsanou F., Kambas A. (2016). Motor Proficiency in Young Children: A Closer Look at Potential Gender Differences. SAGE Open.

[61] Navarro-Patón R., Canosa-Pasantes F., Mecías-Calvo M., Arufe-Giráldez V. (2024). Is It Possible to Improve Motor Competence through a Structured Balance Bike Program in Preschool Children Aged 3 to 6 Years?. Sports.

[62] Costa H.J.T., Abelairas-Gomez C., Arufe-Giráldez V., Pazos-Couto J.M., Barcala-Furelos R. (2015). Influence of a physical education plan on psychomotor development profiles of preschool children. J. Hum. Sport Exerc..

[63] Battaglia G., Giustino V., Tabacchi G., Alesi M., Galassi C., Modica C., Palma A., Bellafiore M. (2020). Effectiveness of a physical education program on the motor and pre-literacy skills of preschoolers from the training-to-health project: A focus on weight status. Front. Sports Act. Living.

[64] Roberts D., Veneri D., Decker R., Gannotti M. (2012). Weight status and gross motor skill in kindergarten children. Pediatr. Phys. Ther..

[65] Katanić B., Veljković A.A., Stojiljković N., Stanković S., Mitić P. (2022). Effects of a 12-week aerobic training program on the cognitive and motor abilities of preschool children. FU Phys. Ed. Sport.

